# Home introduction of baked egg in a cohort of patients in Cork University Hospital in 2011

**DOI:** 10.1186/2045-7022-5-S3-P142

**Published:** 2015-03-30

**Authors:** Aisling Flinn, Jonathan O B Hourihane, Deidre Daly

**Affiliations:** 1 Department of Pediatrics, Cork University Hospital, Cork, Ireland

## Aims

Egg allergy is one of the most common food allergies in children. The prevalence in children is estimated to be approximately 2%. Reaction to raw/lightly cooked egg but tolerance to baked egg is often reported. The natural history of egg allergy is that it usually resolves with time but the rate of resolution is variable. The British Society for Allergy and Clinical Immunology published guidelines in 2011 with regard to the introduction of egg into the diet. The aim of our audit was to assess whether our clinical practice of advising home introduction of baked egg adhered to these guidelines.

## Methods

Clinic letters from 2011 were reviewed by doing a search on our computer data base. The following data was collected: Date of birth, sex, age at diagnosis of egg allergy and type of reaction, specific IgE and skin prick test to egg results, coexisting atopic conditions, history of previous egg challenge, age at which home introduction of baked egg was advised, result of introduction and, if positive, what symptoms were elicited and what treatment was given.

## Results

Total number of patients was 47, 26 males, 21 females. Home introduction was successful in 34/47 (72.3%). Seven patients (14.9%) had a positive reaction 7/47 (14.9%), most of these were mild and required no treatment or treatment with antihistamines. Among 5 patients with co-existing asthma, one had a positive result. Three patients had wheeze as part of their index reaction and two of these had positive challenges.

## Conclusion

Overall, the majority of patients in our cohort who introduced baked egg at home had a safe and successful outcome. It is important when considering candidates for home introduction to take into account the presence of co-existing asthma or multiple food allergies, as well as the type of index allergic reaction.

